# Comparison of Physical Activity Measures Using Mobile Phone-Based CalFit and Actigraph

**DOI:** 10.2196/jmir.2470

**Published:** 2013-06-13

**Authors:** David Donaire-Gonzalez, Audrey de Nazelle, Edmund Seto, Michelle Mendez, Mark J Nieuwenhuijsen, Michael Jerrett

**Affiliations:** ^1^Center for Research in Environmental Epidemiology (CREAL)Barcelona, CataloniaSpain; ^2^Hospital del Mar Research Institute (IMIM)Barcelona, CataloniaSpain; ^3^Spanish Consortium for Research on Epidemiology and Public Health (CIBERESP)Barcelona, CataloniaSpain; ^4^Physical Activity and Sports Sciences Department, Fundació Blanquerna, Ramon Llull UniversityBarcelona, CataloniaSpain; ^5^Center for Environmental Policy, Imperial College LondonLondonUnited Kingdom; ^6^Division of Environmental Health Sciences, School of Public Health, University of CaliforniaBerkeley, CAUnited States; ^7^Department of Nutrition, University of North CarolinaChapel Hill, NCUnited States

**Keywords:** cellular phone, accelerometry, global positioning systems, motor activity, monitoring, physiologic

## Abstract

**Background:**

Epidemiological studies on physical activity often lack inexpensive, objective, valid, and reproducible tools for measuring physical activity levels of participants. Novel sensing technologies built into smartphones offer the potential to fill this gap.

**Objective:**

We sought to validate estimates of physical activity and determine the usability for large population-based studies of the smartphone-based CalFit software.

**Methods:**

A sample of 36 participants from Barcelona, Spain, wore a smartphone with CalFit software and an Actigraph GT3X accelerometer for 5 days. The ease of use (usability) and physical activity measures from both devices were compared, including vertical axis counts (VT) and duration and energy expenditure predictions for light, moderate, and vigorous intensity from Freedson’s algorithm. Statistical analyses included (1) Kruskal-Wallis rank sum test for usability measures, (2) Spearman correlation and linear regression for VT counts, (3) concordance correlation coefficient (CCC), and (4) Bland-Altman plots for duration and energy expenditure measures.

**Results:**

Approximately 64% (23/36) of participants were women. Mean age was 31 years (SD 8) and mean body mass index was 22 kg/m^2^ (SD 2). In total, 25/36 (69%) participants recorded at least 3 days with at least 10 recorded hours of physical activity using CalFit. The linear association and correlations for VT counts were high (adjusted *R* ^*2*^=0.85; correlation coefficient .932, 95% CI 0.931-0.933). CCCs showed high agreement for duration and energy expenditure measures (from 0.83 to 0.91).

**Conclusions:**

The CalFit system had lower usability than the Actigraph GT3X because the application lacked a means to turn itself on each time the smartphone was powered on. The CalFit system may provide valid estimates to quantify and classify physical activity. CalFit may prove to be more cost-effective and easily deployed for large-scale population health studies than other specialized instruments because cell phones are already carried by many people.

## Introduction

Physical inactivity now ranks as the tenth leading cause of premature mortality worldwide [1,2]. Inactivity has increased substantially over the past 15 years [2]. Physical inactivity contributes to the development of major chronic diseases, such as coronary heart disease, stroke, hypertension, colon cancer, breast cancer, Type 2 diabetes, and osteoporosis [3].

Information on physical activity in epidemiological studies is generally obtained by questionnaires and more recently with accelerometers [4]. The latter is becoming the accepted method because of better accuracy and reliability of the physical activity measures [4]. Accelerometers use the acceleration in the subjects’ movements to quantify intensity over short epochs of time. Although an improvement over questionnaires, deploying accelerometers is labor intensive and burdensome to the participant at times and may lead to potential changes in behavior, such as not wearing the accelerometer or increasing the measured behavior [5,6].

To address these problems and take advantage of the increased use and improved technology of smartphones, we developed CalFit [7-10]. CalFit is open-source software that runs on Android smartphones. The system makes use of the accelerometry and Global Positioning System (GPS) sensors that are built into smartphones to record physical activity and the time and location in which an activity occurs. It has the potential to reduce cost and allow for enrollment of more participants because smartphones are now in widespread use in the general population [11]. Smartphones equipped with CalFit could potentially make better physical activity measurements compared to a common accelerometer, particularly because of the addition of GPS measurements that can help researchers better understand the spatial context of activity [12]. Calibration and validation work of CalFit has been conducted thus far only under laboratory conditions [13].

The aim of this research is to study the usability of CalFit software and to assess the validity of its physical activity measures in real world situations by comparing its physical activity measures under free-living conditions with those obtained from a well-known and validated accelerometer, the Actigraph GT3X [14].

## Methods

### Sample

We enrolled volunteers to wear the CalFit phone and a conventional accelerometer for 5 days. Thirty-six participants were recruited by way of emails sent to colleagues from the Centre for Research in Environmental Epidemiology (CREAL) and to friends of colleagues as part of a larger study based on active travel behaviors. Inclusion criteria were to live and work or study in Barcelona, to live more than 10 minutes walking distance from the workplace or school, and be able to ride a bike for at least 20 minutes. Volunteers who met the eligibility requirements were enrolled in the study after an information session in which they were provided with details on study objectives and procedures. The field study took place from November 2011 to February 2012.

Our study protocol was approved by the Ethics Committee of Hospital del Mar Research Institute, and written informed consent was obtained from all the participants.

### Instruments

**Figure 1:**
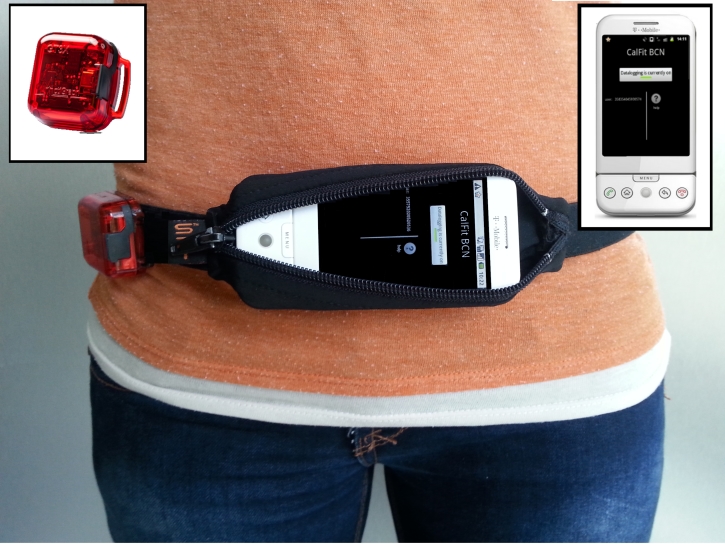
Set of devices that were worn during 5 consecutive days.

Each participant was given an Actigraph GT3X accelerometer [15] and a smartphone fitted with the CalFit application (see Table 1). The devices were worn during 5 consecutive days on a belt attached to the waist (see Figure 1). Participants were instructed to remove devices only when performing aquatic activities or sleeping, or when necessary to charge the smartphone battery.

**Table 1:** Characteristics of CalFit and Actigraph GT3X.

Characteristics	Google G1 with CalFit	Actigraph GT3X
Size	11.7×5.6×1.7 cm	3.8×3.7×1.8 cm
Weight	158 g	27 g
Placement	Frontal mean point between both anterior superior iliac spines	Anterior superior iliac spine of the right hip
Sample rate	10 Hz	30 Hz
Data storage	16 GB	16 MB
Battery life	18 hours	31 days
Accelerometer sensor	AK8976A triaxial accelerometer (Asahi Kasei Microsystems, Japan)	ADXL335 triaxial accelerometer (Analog Devices, Norwood, MA)
Registered range of acceleration	±2.8*g*	±3*g*
Outcomes (measured)	Acceleration of the 3 axes	Acceleration of the 3 axes
Outcomes (estimated)	Not wearing; energy expenditure and duration of physical activity	Not wearing; standing, sitting, and lying; energy expenditure and duration of physical activity

### Data Treatment

Data from both devices were summarized to 1-minute intervals. We merged data streams from both accelerometers identifying the time alignment that yielded the highest association (adjusted *R* ^*2*^) between the 2 vertical (VT) axis measures, within a maximum of 5-minute differences in time. To maximize the comparability [16], the intensity of physical activity measured in metabolic equivalents (METs) by both devices was calculated according to the equation of Freedson et al [17], which uses a linear function based on vertical axis counts to produce their estimates: ActiGraph GT3X METs=1.439008 + (0.000795 * VT counts/min). Because VT axis measures were recorded by each instrument with different units (counts from Actigraph GT3X versus *g*-force from CalFit), we first developed a linear regression between the 2 vertical measures to convert the CalFit *g*-force/min into counts/min, leading to the following adaptation to the Freedson equation for estimating METs from smartphone data: CalFit METs=1.2907087 + (0.4141791 * VT *g*/min).

The accelerometer nonwear intervals were defined as episodes of at least 40 consecutive minutes of 0 counts and below 0.3*g* in vertical axis for Actigraph GT3X and CalFit, respectively. The latter threshold was established after analyzing CalFit nighttime measurements. The American College of Sports Medicine considers having at least 3 days with at least 10 hours of recorded activity as a valid assessment of physical activity [18]. We measured the usability of CalFit and of Actigraph GT3X, understood as the ease of use to reach valid assessment of physical activity, in 4 ways: (1) number of subjects with valid assessments of physical activity (previous definition); (2) number of recorded days per participant (ie, reflecting participants’ ability to keep CalFit turned on); (3) total recorded time per participant (ie, reflecting participants’ ability to keep batteries charged); and (4) percent wearing time from total recorded time (ie, reflecting participants’ ability to wear CalFit).

Physical activity was defined as any minute with intensity equal or greater than 1.5 METs. Physical activity was partitioned into light, moderate, and vigorous levels of physical activity following the conventional cutoffs of 3 and 6 METs. The main summary measures of physical activity were vertical axis counts, and duration and intensity of physical activity.

### Analysis

Participants and physical activity characteristics are presented as number (percentage) for categorical variables, mean (SD) for continuous variables with normal distribution, or median (interquartile range, IQR) for continuous variables with non-normal distribution.

The comparison between CalFit and Actigraph GT3X was conducted using several approaches. First, to assess differences on usability as defined above, we performed a Kruskal-Wallis rank sum test (difference of medians components of usability). Second, the correlation and association between the vertical axis measures during coinciding time periods were assessed through a Spearman correlation and linear regression, respectively. Third, the agreement in the main summary measures of physical activity, as previously defined and during coinciding time periods, was studied using Lin’s concordance correlation coefficient (CCC) [19] and Bland-Altman plots. The CCC can be conceptualized as the ratio of between-subject variance to total variance [20]. In other words, it provides a measure of the percentage of differences attributable to the participants, and its complement (1-CCC) gives the percentage of differences attributable to the method (ie, CalFit vs Actigraph GT3X). The bias between instruments was evaluated using a linear regression analysis between the differences (CalFit-Actigraph GT3X) and the mean, as 0.5*(CalFit-Actigraph GT3X), of the 2 physical activity measures, considering the bias to be significant when the confidence interval of the coefficient did not contain the value zero. Both regression coefficient and regression line of bias were also plotted into Bland-Altman plots indicated with red letter and line, respectively.

As a sensitivity analysis, previous comparisons were also performed during coinciding days with at least 10 hours of recorded activity, without control of the coinciding time periods, to test the influence of nonmeasured periods on physical activity agreement. All analyses were conducted using R-2.14.1 2011 (The R Foundation for Statistical Computing).

## Results

The sample consisted of 36 participants, most of which were women (23/36, 64%), with a mean age of 30.9 years (SD 7.9), and mean body mass index of 22.2 kg/m^2^ (SD 2.4) (Table 2). Approximately 83% (30/36) were of Spanish nationality, 92% (33/36) had high school or greater education, and 50% (18/36) earned more than €2000 per month.

### CalFit Usability

Of 180 possible days for recorded data, 19 were missing from CalFit and 8 from the Actigraph GT3X. During recorded days, there was a significant difference between the median time recorded: 22 hours for CalFit and 24 hours for Actigraph GT3X (Table 3). Also, there were differences for the percent of wear time between CalFit and Actigraph GT3X (52% vs 59%) (Table 3). The median number of days with at least 10 wearing hours was 3 and 5 for CalFit and Actigraph GT3X, respectively.

The main reasons for failed CalFit data collection among the 11 subjects who recorded less than 3 valid assessment of physical activity were: (1) 6 lost an average of 2 days of recording because CalFit was inadvertently turned off, (2) 2 had problems with phone battery life and their daily routine, and (3) 3 did not wear the phone.

**Table 2:** Sociodemographic and physical characteristics of all participants (N=36).

Sample characteristics		Participants
Age (years), mean (SD)		31 (8)
**Gender, n (%)**		
	Male	13 (36)
BMI (kg/m^2^), mean (SD)		22 (2)
**Educational level, n (%)**		
	More than high school	33 (92)
	Less than high school	3 (8)
**Nationality, n (%)**		
	Spanish	30 (83)
	Others	6 (17)
**Monthly income (€)**		
	More than 2000	18 (50)
	Less than 2000	18 (50)
**Working status, n (%)**		
	Working	32 (89)
	Studying	4 (11)

**Table 3:** Comparison of usability characteristics between Actigraph and CalFit.

Characteristic	Actigraph GT3X	CalFit	*P* value
Days recorded (day), median (IQR)	5 (5-5)	5 (4.8-5.0)	.03
Recorded time (min), median (IQR)	7200 (7200-7200)	6474 (4635-7068)	<.001
Wearing time (min), median (IQR)	4109 (3735-4373)	2938 (2269-3652)	<.001
Time coincident (min), median (IQR)	2825 (2110-3556)		
Recorded time within recorded days (hour/day), median (IQR)	24 (24-24)	22 (20-24)	<.001
Worn time within recorded days (hour/day), median (IQR)	14 (12.5-15)	11 (10-13)	<.001
Percent of worn time on recorded time within recorded days (%),median (IQR)	58.5 (53-63)	51.6 (46-58)	.03
Number of days with at least 10 wearing hours (day), median (IQR)	5 (4-5)	3 (2-4.2)	<.001
Participants with valid assessment of physical activity, n (%)	34 (94)	25 (69)	<.001

### Validity of Physical Activity

**Figure 2:**
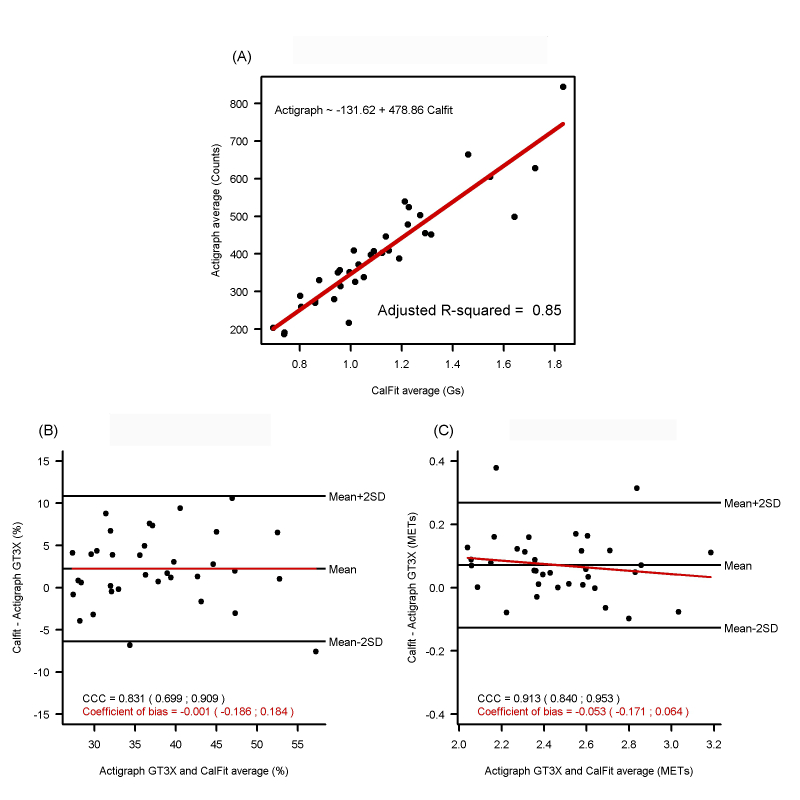
Agreement between CalFit and Actigraph GT3X in vertical axis, duration, and energy expenditure in physical activity within the coinciding measurement time periods. (A) accelerometer vertical axis measures, (B) duration in physical activity, and (C) intensity of physical activity.

The linear regression and correlation analysis for average vertical (VT) axis measures from both devices during coinciding wear-time periods showed a high association (adjusted *R* ^*2*^=0.85; Spearman correlation coefficient .932, 95% CI 0.931-0.933) (Figure 2, part A). During coinciding time periods (mean time/day, 2600 min), the mean difference between Actigraph GT3X and CalFit for the duration of active time (>1.5 METs) was 2.24% (95% CI 0.76-3.72) and for intensity of physical activity was 0.07 METs (95% CI 0.04-0.1) (Figures 2, parts B and C). The comparison for both the duration and intensity of physical activity showed that the variability attributable to the measurement method (which is the complementary to the ratio of between-subject variance to total variance) was less than 20% (Figures 2, parts B and C). There was no association between difference and average of both measures neither for duration (*P*=.55) nor for intensity (*P*=.22) of physical activity.

### Validity of Physical Activity Through the Different Intensity Thresholds

**Figure 3:**
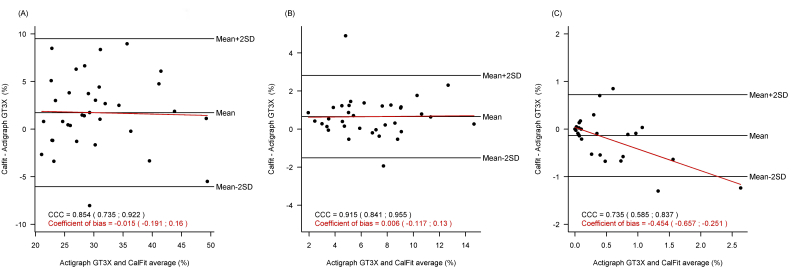
Agreement between CalFit and Actigraph GT3X for duration of light, moderate, and vigorous physical activity within the coinciding measurement time periods. (A) duration of light physical activity, (B) duration of moderate physical activity, and (C) duration of vigorous physical activity.

**Figure 4:**
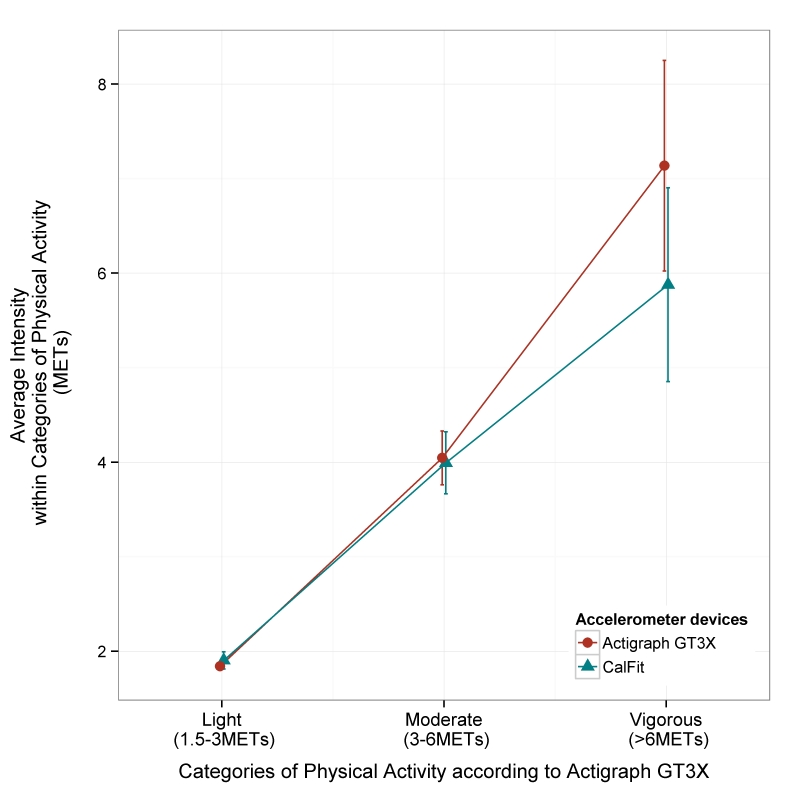
Comparison of average intensity recorded by CalFit and Actigraph GT3X within light, moderate, and vigorous physical activity identified by Actigraph.

The comparison of measures of light, moderate, and vigorous physical activity showed that less than 30% of the variability was attributable to the method of measurement (Figure 3). In contrast to light and moderate physical activity, the CalFit measures of vigorous physical activity showed a tendency to underestimate the duration in vigorous physical activity as activity levels increased (*P*=.01) compared to the Actigraph GT3X measure (Figure 3, part C). Figure 4 shows there was a significant underestimation in the intensity recorded by CalFit when participants performed vigorous activity according to Actigraph GT3X (CalFit: mean 5.9, SD 1.0; Actigraph: mean 7.1, SD 1.1; *P*<.001).

### Sensitivity to Measurement Period

**Figure 5:**
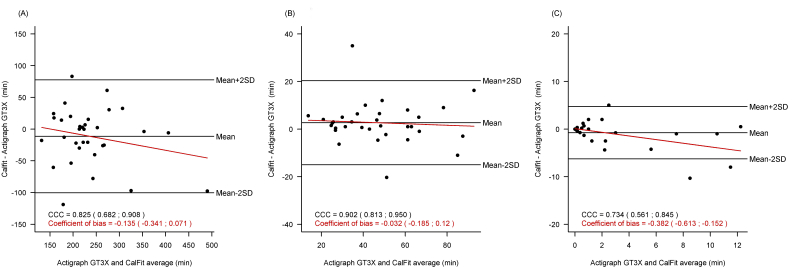
Agreement between CalFit and Actigraph GT3X during light, moderate, and vigorous physical activity within the coinciding days with at least 10 hours of recorded activity. (A) duration of light physical activity per day, (B) duration of moderate physical activity per day, and (C) duration of vigorous physical activity per day.

Depending on the time inclusion criteria selected, the average difference between Actigraph GT3X and CalFit during light physical activity changed from a small but significant overestimation of 1.7% (95% CI 0.4-3.1) for coinciding time periods to a nonsignificant underestimation of –11.5 min (95% CI –27 to 4.3) for coinciding valid days (Figure 3, part A vs Figure 5, part A). There were no differences in duration of moderate and vigorous physical activity across time inclusion criteria (Figures 3, parts B and C vs Figures 5, parts B and C).

## Discussion

### Principal Results

This study assessed the usability and validity of CalFit software in a group of free-living volunteers. We compared CalFit to physical activity measures with those obtained from the Actigraph GT3X. The several approaches used to assess the properties of the CalFit showed that (1) there is a strong association between vertical axis measures from both devices; (2) the measures of duration and energy expenditure in overall, light, and moderate physical activity were highly concordant between devices, whereas vigorous physical activity was underestimated; (3) CalFit had lower usability compared to Actigraph GT3X resulting in a lower proportion of participants with valid assessment of physical activity; and (4) sensitivity analysis that compared the agreement within coinciding time periods to the agreement within coinciding days with at least 10 hours of recorded activity showed that the disparities in wearing-time periods between devices did not contribute to any significant bias into the measured validity.

### Comparison With Prior Work

To our knowledge, this is the first study to compare accelerometer use on smartphones to measure physical activity with a currently well-validated instrument [12]. Previous research on physical activity assessment with mobile phones has shown that they are a useful tool to perform interventions [21] and are helpful for activity recognition [22-24]. In addition to these advantages, using smartphones for physical activity research opens up opportunities for reaching large numbers of participants at a relatively low cost [9]. The acceptance and usability of smartphones to measure physical activity on free-living conditions was previously unknown. Here, we showed that 25 of 36 (69%) participants who used CalFit recorded at least 3 valid days, which is the minimum recommended to assess daily physical activity [25]. The greatest weakness in CalFit usability was loss of data because of the phone turning off and not having CalFit restart when the phone was powered back on (50% of the missing data). In the current version of CalFit, this problem has been solved by automatically restarting CalFit each time the phone is turned on. The second weakness was the battery life (recorded time and wearing time), which was responsible for the other half of the missing data. The difference in wearing time with the accelerometer was partly because of participants having to charge the smartphone during waking hours (as they were instructed to do). Since conducting our study, we have found that newer generation smartphones have improved battery life, and current field tests indicate that CalFit is recording for longer durations without charging [26].

This is also the first study to compare the validity of the vertical axis measures and to use the same algorithm for estimating physical activity in 2 different instruments. The association of the vertical axis measures between the 2 tools was high (adjusted *R* ^*2*^ 0.86; correlation coefficient .932; 95% CI 0.931-0.933), which is within the range of the literature comparing different models of Actigraph [27]. The concordance found in duration and energy expenditure in physical activity measures of CalFit and Actigraph GT3X showed that the measures from CalFit and Actigraph GT3X are interchangeable (less than 20% of the variability is attributable to differences between instruments).

Concordance in physical activity measures across different definitions of time inclusion criteria showed that the results remained constant despite the shorter wearing time of CalFit. This suggests that the time charging the smartphone or the shorter battery life did not have a significant influence on the final measures. There was also a statistically significant bias toward underestimation in measures of vigorous physical activity estimated by CalFit compared to Actigraph GT3X. This may be partially explained by the fact that we used average VT measures instead of all measures per minute per participant and because we assumed a linear relationship between acceleration forces from smartphone and counts from Actigraph GT3X.

### Strengths

One of the main strengths of this study is the use and testing under free-living conditions. Participants maintained their daily routines, which is difficult to replicate in controlled environments. Another strength was the use of the concordance measures for quantifying physical activity in addition to the commonly used correlations. A third was using the Freedson algorithm of physical activity for both instruments, which is a valid algorithm for the different Actigraph models (CSA 1764, GT1M, and GT3X) [28] that maximized the comparability between the instruments [16]. Furthermore, because we used a first-generation smartphone, our findings can be generalized and expected to be better for the latter generations of smartphones as a consequence of hardware evolution. Also, future versions on CalFit will be developed for Android and iPhone platforms.

The validation of smartphone accelerometry-based energy expenditure has implications for both epidemiologic research on physical activity as well as for the growth of the practice of medicine and public health by mobile applications (mHealth applications). Beyond the current CalFit application, which is focused on unobtrusive sensing of physical activity, may be novel mHealth smartphone applications that not only record physical activity, but attempt to intervene upon behavior [11,21]. For example, future use of smartphones may allow for recognition of patterns of physical activity to better tailor interventions to personal baselines and goals. Additionally, future interventions may employ other aspects of smartphone technology (eg, call, text messaging, and Internet communication capabilities) to combine physical activity monitoring with motivational social interactivity [21]. There are many possibilities for creative uses of smartphones, and the research presented here provides a foundation for better understanding the energy expenditure estimates from this technology.

The CalFit smartphone system has several advantages over conventional accelerometers because of geolocation information both from cell phone towers and Wi-Fi networks and from GPS satellites. This geolocation will allow us to improve the current physical activity algorithm by including information such as velocity of displacements, topographical challenges faced by participants (stairs, slopes), and the environments (home, work) where physical activity occurred. Furthermore, this tool also allows assessments of how the built and natural environment may affect behavior or lead to other exposures. Our research group has begun to demonstrate some of these advantages with the same participants by characterizing where the physical activity was done and quantifying the amount of pollution inhaled by participants in these environments [29].

### Limitations

One limitation of the present study was the use of a convenience sample of 36 participants with a high educational level to assess CalFit usability. However, this design has been efficient in detecting the problems in usability. Further work needs to be conducted in the population at large. Second, the use of the Actigraph GT3X accelerometer as a gold standard could be seen as a limitation, but it is the reference tool for assessing physical activity in real life for 5 days and has well-established validity [30]. The use of an algorithm for the METs estimation that only takes into account the vertical axis of the accelerometers is a limitation that we could not avoid because there are currently no published Actigraph GT3X algorithms using the 3 axes of the accelerometer [31,32]. Finally, our definition of CalFit wearing time was an operational definition and should not be considered as a reference until it is tested in studies specifically designed for this purpose.

### Conclusions

Compared to the current gold standard instrument for population studies, the smartphones fitted with CalFit supply useful and valid estimates for quantifying and classifying physical activity under free-living conditions. Although user compliance for CalFit was lower than with the Actigraph GT3X, this difference would likely diminish if participants were allowed to load CalFit onto their existing smartphones, which will be feasible in the future. Such deployment would provide a cost-effective approach for large epidemiological studies and mHealth applications that rely upon measured physical activity.

## Abbreviations

CCC: concordance correlation coefficient
GPS: Global Positioning System
MET: Metabolic Equivalent of Task
VT: vertical
